# What WGS Reveals about *Salmonella enterica* subsp. *enterica* in Wildlife in Germany

**DOI:** 10.3390/microorganisms9091911

**Published:** 2021-09-09

**Authors:** Laura Uelze, Angelina Bloch, Maria Borowiak, Mirjam Grobbel, Carlus Deneke, Matthias Fischer, Burkhard Malorny, Michael Pietsch, Sandra Simon, István Szabó, Simon H. Tausch, Jennie Fischer

**Affiliations:** 1 Department of Biological Safety, German Federal Institute for Risk Assessment (BfR), Max-Dohrn-Str. 8-10, 10589 Berlin, Germany; Laura.Uelze@bfr.bund.de (L.U.); Angelina.Bloch@bfr.bund.de (A.B.); Maria.borowiak@bfr.bund.de (M.B.); Mirjam.grobbel@bfr.bund.de (M.G.); Carlus.Deneke@bfr.bund.de (C.D.); Matthias.Fischer@bfr.bund.de (M.F.); Burkhard.Malorny@bfr.bund.de (B.M.); Istvan.Szabo@bfr.bund.de (I.S.); simon.tausch@bfr.bund.de (S.H.T.); 2 Unit for Enteropathogenic Bacteria and Legionella (FG11)/National Reference Centre for Salmonella and Other Bacterial Enteric Pathogens, Robert Koch Institute (RKI), Burgstr. 37, 38855 Wernigerode, Germany; PietschM@rki.de (M.P.); simons@rki.de (S.S.)

**Keywords:** *Salmonella enterica* subsp. *enterica*, whole-genome sequencing, wildlife

## Abstract

The aim of this study was to gain an overview of the genetic diversity of *Salmonella* found in wildlife in Germany. We were particularly interested in exploring whether wildlife acts as a reservoir of certain serovars/subtypes or antimicrobial resistance (AMR) genes. Moreover, we wanted to explore the potential of *Salmonella* in spreading from wildlife to livestock and humans. To answer these questions, we sequenced 260 *Salmonella enterica* subsp. *enterica* isolates sampled between 2002 and 2020 from wildlife across Germany, using short-read whole genome sequencing. We found, consistent with previous findings, that some *Salmonella* sequence types are associated with certain animal species, such as *S.* Choleraesuis ST145 with wild boar and *S.* Enteritidis ST183 with hedgehogs. Antibiotic resistance was detected in 14.2% of all isolates, with resistance against important WATCH group antibiotics present in a small number of isolates. We further found that wildlife isolates do not form separate phylogenetic clusters distant to isolates from domestic animals and foodstuff, thus indicating frequent transmission events between these reservoirs. Overall, our study shows that *Salmonella* in German wildlife are diverse, with a low AMR burden and close links to *Salmonella* populations of farm and food-production environments.

## 1. Introduction

Non-typhoidal *Salmonella* are important zoonotic bacteria that infect a wide range of domestic and wild animal species and cause salmonellosis in humans [1]. The main reservoir of *Salmonella* is food-producing animals. However, *Salmonella* can persist under various conditions and are also able to survive outside the animal or human gut for longer time periods [2,3]. Due to their human health impact, most countries have established national monitoring and control programs on the occurrence of *Salmonella* in livestock [4]. Equally, most investigations focus on *Salmonella* in livestock populations and food products. Infection of humans due to wildlife contact is reported only rarely, and thus studies on *Salmonella* in wildlife are scarce. Available studies often focus on specific serovars occurring in wildlife, while other studies have reported on the increased risk of children due to their greater exposure to the natural environment [5,6,7,8].

Furthermore, most available studies are based on classical typing methods such as multilocus sequence typing (MLST), pulsed field gel electrophoresis (PFGE) and phage typing. However, with the recent adoption of Next Generation Sequencing (NGS) for *Salmonella* typing and outbreak analysis, the availability of whole genome sequencing data allows a deeper insight into the characteristics of *Salmonella* isolates from wildlife populations, their transmission and natural reservoirs [9]. Studying *Salmonella* from wildlife can enhance our understanding of their behavior in natural and urban surroundings apart from food-production systems. Importantly, analysing *Salmonella* isolates from wildlife for the presence of certain AMR genes or AMR-carrying plasmids yields information about the extent of the environmental pollution [10]. Furthermore, as *Salmonella* outbreak investigations are increasingly based on WGS data, wildlife *Salmonella* isolates with close genetic similarity to human isolates are sporadically detected, raising questions about the transmission routes between different human and animal populations and reservoirs. Finally, the COVID-19 pandemic has further emphasized the need for the investigation of infectious agents in wildlife and the real extent of wildlife—human transmissions [11].

Here, we present the WGS analysis of a convenience sample of 260 wildlife *Salmonella enterica* isolates, collected between 2002–2020 by the German National Reference Laboratory for *Salmonella* (NRL-*Salmonella*). We characterized draft assemblies and screened for the presence of AMR genes and plasmids. We applied cgMLST and SNP analysis to compare genome sequence data from wildlife isolates with a large database of sequencing data of *Salmonella* isolates from humans, food and farm animals. We examined whether there are certain clones circulating in German wildlife populations that are linked to specific reservoirs. We further investigated whether isolates from wildlife are also found in the livestock and human sector, as well as in other (European) countries. Finally, we studied phylogenetic relationships of mixed-matrices clusters, in order to explore the potential transmission links between wildlife and humans.

## 2. Materials and Methods

### 2.1. Sample Selection

The German National Reference Laboratory provided all wildlife isolates for *Salmonella* (NRL-*Salmonella*). We combined three different strategies for the compilation of the data set: Firstly, we included 30 wildlife isolates, which were sequenced previously, as part of routine *Salmonella* surveillance (published under BioProject PRJEB31846). Secondly, we included 64 wildlife *S.* Choleraesuis isolates sequenced in 2020 in the frame of the GenoSalmSurv project [4]. Thirdly, we applied the R function *sample_n* to randomly select wildlife *Salmonella enterica* isolates from the large NRL-*Salmonella* collection of previously not-sequenced isolates. We aimed to draw an equal distribution across sampling years, federal states and host species where possible (i.e., where isolates were available). As the collection contains a very large number of isolates from mammalian wildlife, we decided to only include isolates from two separate time windows (2007–2010 and 2016–2020), rather than across the entire time span. For wild birds, a smaller number of isolates was available, therefore a larger time window (2007–2020) was considered. For greater feasibility, as the database contains a large collection of pigeon-derived isolates, we limited the number of samples obtained from pigeons to one sample per year. Following these parameters, 166 samples were selected and subsequently sequenced for the purpose of this study. In total, we obtained a data set of Illumina short-read sequencing data for 260 wildlife isolates. At each selection step, we took care to only include isolates obtained from truly free-living native and invasive (e.g., raccoon) wildlife.

### 2.2. Comparison Data Sets

For comparison with isolates from farm animals and foodstuff, we employed an in-house collection of short-read whole-genome sequencing data of *Salmonella* isolates (*n* = 3824 (17 March 2021)), obtained by the NRL-*Salmonella* for the purpose of *Salmonella* surveillance and outbreak investigations. The database encompasses mainly isolates from farm animals (mostly chicken and pig) and food (mostly eggs and meat).

In addition, we included human and non-human isolates collected in 2020, in the frame of the ongoing real-time surveillance project GenoSalmSurv [4] (*n* = 1896 (5 March 2021)).

We further included short-read whole-genome sequencing data of human clinical *Salmonella* isolates, which were part of outbreak investigations between 2018 and 2020 (*n* = 101 (22 February 2021)). These were provided by the National Reference Centre for *Salmonella* and other Bacterial Enteric Pathogens (NRC-*Salmonella)* located at the Robert Koch Institute (RKI). Moreover, human isolates from the ENA database were included if an involvement in European outbreaks was found.

For comparison with international data, we included short-read whole-genome sequencing data from *Salmonella* isolates obtained from wildlife investigated in other studies. All external data were processed identically to in-house sequencing data, as described below.

### 2.3. Whole-Genome Sequencing and Assembly

Bacteria were cultivated on lysogeny broth (LB) agar. A single colony was inoculated in liquid LB and cultivated under shaking conditions (180–220 rpm) at 37 °C for 14–16 h. Genomic DNA was extracted from liquid cultures using the PureLink^®^ Genomic DNA Mini Kit (Invitrogen, Carlsbad, CA, USA). Sequencing libraries were prepared with the Nextera™ XT DNA Library Preparation Kit or the Nextera™ DNA Flex Library Preparation Kit (Illumina, San Diego, CA, USA) according to the manufacturer’s protocol. Paired-end sequencing was performed on the Illumina MiSeq™ benchtop sequencer (Illumina, San Diego, CA, USA) using the MiSeq™ Reagent Kit v3 (600-cycle) or on the Illumina NextSeq™ 500 benchtop sequencer (Illumina, San Diego, CA, USA) using the NextSeq™ 500/550 Mid Output Kit v2 or v2.5 (300-cycle) (Illumina, San Diego, CA, USA). Raw reads were trimmed and *de novo* assembled with the AQUAMIS pipeline v1.3.9 [12], which implements fastp v0.19.5 for trimming [13] and shovill v1.1.0 (https://github.com/tseemann/shovill; accessed on 8 September 2021) for assembly.

### 2.4. Bacterial Characterization

Bacterial isolates were serotyped according to the White–Kauffmann–Le Minor scheme by slide agglutination with O- and H-antigen specific sera (Sifin Diagnostics, Berlin, Germany) before they underwent whole genome sequencing.

Draft genome assemblies were analysed with the Bakcharak pipeline v2.0 (git version 1.0.0-77-g5b31a01) (https://gitlab.com/bfr_bioinformatics/bakcharak; accessed on 8 September 2021), which implements, among other tools, ABRicate v1.0.1 for antimicrobial resistance and virulence factor screening (https://github.com/tseemann/abricate; accessed on 8 September 2021), together with the PlasmidFinder database for plasmid detection [14], mlst v2.19.0 (https://github.com/tseemann/mlst; accessed on 8 September 2021) for ST typing, SISTR v1.0.2 [15] for *in silico Salmonella* serotyping and Prokka v1.13 [16] for gene annotation. Platon [17] was used to determine whether an AMR gene-harbouring contig was classified as part of a plasmid or the chromosome.

### 2.5. Testing of Minimum Inhibitory Concentration (MIC) of Isolates

Phenotypic resistance properties were determined using the broth microdilution method according to CLSI guidelines (M07-A10 and CLSI M45-A). Phenotypic AMR was assessed according to epidemiological cut-offs provided by the European Committee on Antimicrobial Susceptibility Testing (EUCAST) and fixed in Commission Implementing Decision 2013/652/EC [18]. The test panel included antimicrobial agents Gentamicin, Chloramphenicol, Cefotaxime, Ceftazidime, Nalidixic acid, Ciprofloxacin, Ampicillin, Colistin, Sulfamethoxazole, Trimethoprim, Tetracycline, Azithromycin, Meropenem and Tigecycline with test-concentration ranges and evaluation criteria previously described [19].

### 2.6. Phylogenetic Analysis

cgMLST allele calling was conducted with the chewieSnake pipeline v1.2 [20], which implements chewBBACA v2.0.16. The cgMLST scheme for *Salmonella enterica* was derived from Enterobase (http://enterobase.warwick.ac.uk/species/senterica/download_data; accessed on 8 September 2021). SNP calling was conducted with the snippySnake pipeline v1.0 [21] which implements snippy v4.1.0 (https://github.com/tseemann/snippy; accessed on 8 September 2021) for variant calling. A maximum-likelihood based phylogenetic tree was inferred with IQ-TREE v1.6.12 [22] from the core SNPs. Phylogenetic trees were visualized with iTOL v5 [23].

The following references were used for SNP calling: for *S.* Choleraesuis ST145: NZ_CM001062.1 (*S.* Choleraesuis str. SCSA50 isolate A50); for *S.* Enteritidis ST11: NZ_CP025554.1 (*S.* Enteritidis strain ATCC BAA-708); for *S.* Typhimurium ST128: NC_022544.1 (*S.* Typhimurium str. DT2), for *S.* Typhimurium ST19: NZ_CP020922.1 (*S.* strain 16A242).

As no complete genome sequence was available from the public repositories for serovar *S.* Ball, a whole genome shotgun sequence of *S.* Ball strain PNUSAS002191 (Accession: AAONAJ010000001-AAONAJ010000017) was used as a reference. Similarly, for lack of a complete genome sequence for *S.* Enteritidis ST183, a draft assembly of 19-SA03305-0 was used as a reference.

## 3. Results

### 3.1. Analysis of the Wildlife Dataset and Serovar Distribution

**Figure 1:**
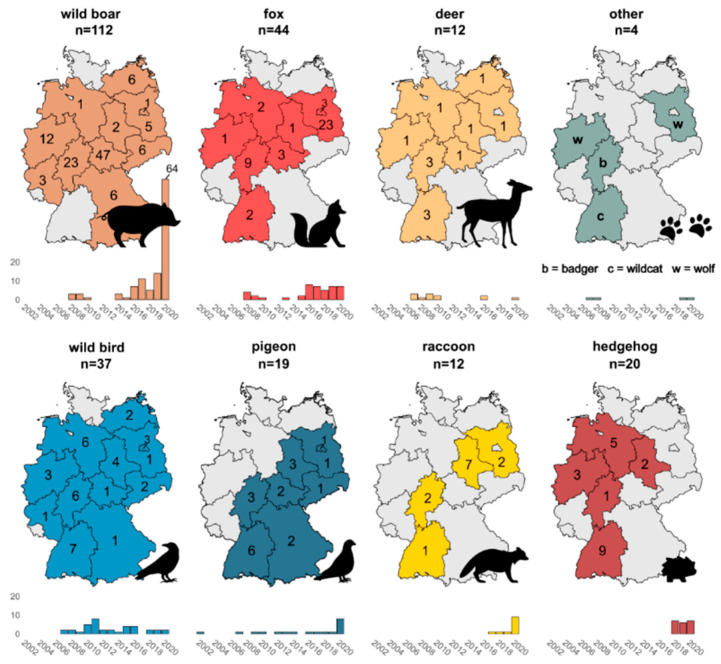
Geographic and temporal distribution of the investigated wildlife *Salmonella enterica* isolates.The figure gives a visual overview of the geographical origin of the investigated wildlife *Salmonella enterica* isolates, grouped by host species, with the total number of samples per host species displayed above each graph. The German federal states that were sampled are highlighted in colour with the total number of samples obtained from each federal state indicated. A timeline below each graph indicates the sampling years when isolates were obtained.

In total, we investigated a dataset of 260 wildlife isolates, obtained between the years 2002 and 2020 from a variety of wildlife species across Germany (Figure 1 and Supplementary File S1). The majority of isolates were obtained from mammalian species (*n* = 204), with most isolates originating from wild boar (*n* = 112) and red foxes (*n* = 44). Other mammalian species included deer (*n* = 12), hedgehog (*n* = 20), raccoon (*n* = 12), badger (*n* = 1), wildcat (*n* = 1) and wolf (*n* = 2).

In addition, the dataset contains 56 isolates from avian species, with 19 pigeon-derived isolates and 37 isolates obtained from various other types of wild birds (starling, tit, magpie, duck, swan, stork, heron, crane, crow, raven, owl, falcon, buzzard, sparrow hawk, pheasant, grouse and quail).

As seen from Figure 1, geographical and temporal distribution of the isolates is uneven and varies by wildlife species. These spatial and temporal biases are a result of the employed sample selection strategy.

**Figure 2:**
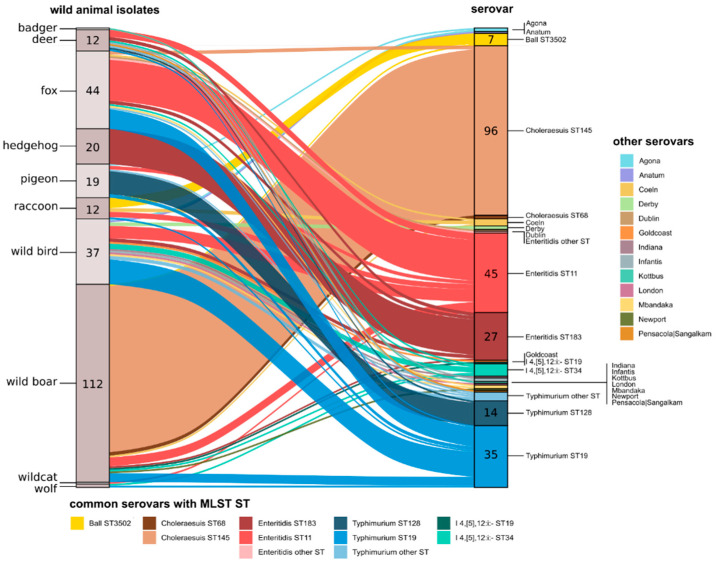
Relationships between the wildlife host species and serovar/MLST sequence types of the *Salmonella enterica* isolates.The alluvial plot visualizes the relationships between host species and serovar/MLST sequence types of the wildlife *Salmonella enterica* isolates. The left y-axis represents the wildlife host species with the number of isolates obtained from each species indicated. The right y-axis represents the serovar (major serovars are further split by ST) with the number of isolates for each serovar/ST indicated. The alluvia are coloured by serovar/ST.

*In silico* serotyping of all isolates revealed an unequal distribution of serovars across the different wildlife species (Figure 2). The most commonly found serovars were *S.* Choleraesuis, *S.* Enteritidis and *S.* Typhimurium. Interestingly, within these serovars, different sequence types were found to be associated with different animal species. For example, all hedgehog isolates belonged to *S.* Enteritidis ST183, while *S.* Typhimurium ST128 accounted for the majority of pigeon isolates. Wild boar isolates could generally be attributed to *S.* Choleraesuis ST145. While some animal species were uniform in the detected serovars (hedgehogs, pigeons), a great number of different serovars was detected for foxes, deer and wild bird isolates. The largest diversity of serovars was found in the 37 wild bird isolates, reflecting the diverse types of wild bird species (*n* = 18) encompassed in this group. Wild bird isolates also accounted for many of the serovars considered rare or less frequently detected, such as *S.* Anatum, *S.* Derby, *S.* Goldcoast, *S.* Indiana, *S.* London and *S.* Mbandaka. The three isolates obtained from the predator species wolf and wildcat were serotyped as *S.* Typhimurium (mono- and biphasic) and *S.* Enteritidis, respectively.

### 3.2. Phenotypic and Genotypic Antimicrobial Resistance in Wildlife Isolates

No genotypic resistance determinants were detected in 85.8% (223/260) of the investigated wildlife isolates. The set of AMR genes detected in the remaining 37 isolates is described in Table 1. Results were confirmed by phenotypic antimicrobial resistance screening for a selection of isolates listed in Supplementary File S1. AMR genes were detected in the serovars *S.* Mbandaka (1/2, 50%), I 4,[5],12:i:-. (7/8, 87.5%), *S.* Choleraesuis (15/98, 15.3%), *S.* Typhimurium (7/54, 13%) and *S.* Enteritidis (3/73, 4.1%). In addition, all *S.* Agona (*n* = 2) and all *S.* Derby (*n* = 2) isolates carried AMR genes. Resistance against aminoglycoside (streptomycin) and folate pathway antagonist antimicrobials (sulfamethoxazole, trimethoprim) were the most frequently detected. We also found a number of isolates with resistance against beta-lactam antimicrobials (ampicllin, amoxicillin), (fluoro-)quinolone (ciprofloxacin, nalidixic acid), tetracycline and fosfomycin. A small number of isolates carried resistance genes against macrolide and phenicol antimicrobials (chloramphenicol, florfenicol). The prevalence of AMR genes in isolates originating from different wildlife species varied, with the highest prevalence found in wild birds (9/37, 24.3%) and foxes (8/44, 18.2%). Similarly, isolates from wild birds and foxes exhibited the highest number of AMR genes per isolate, with individual isolates possessing resistance against up to five different classes of antimicrobials. The monophasic *S.* Typhimurium isolate obtained from a wolf also carried a considerable number of AMR genes, conferring resistance against four different classes of antimicrobials. No resistance genes were observed in isolates obtained from hedgehog, raccoon, wildcat and badger.

### 3.3. Detailed Analyses and Phylogenetic Study of Serovar S. Choleraesuis var. Kunzendorf, ST145 in Wild Boar

*S.* Choleraesuis var. Kunzendorf, ST145, was the dominant serovar isolated from wild boar. Of the 112 isolates obtained from wild boar, 96 isolates were serotyped as serovar *S.* Choleraesuis (85.7%). All but two of these isolates were of ST145 (the other two were ST68). In addition, ST145 was also detected in two isolates obtained from foxes (13-SA01070-0 and 20-SA01308-0). For a phylogenetic analysis, we furthermore included all remaining available *S.* Choleraesuis sequences (which were all ST145) from our in-house database. At the time of the analysis, our database contained 40 isolates from farm animals (domestic pigs *n* = 38, cattle *n* = 2), as well as three food isolates; one each from pork liver, wild boar liver and wild boar meat.

For comparison with international data, we included short-read data from 30 isolates with ST145 obtained from wild boar in Italy (PRJEB27935) [24] and eight isolates with ST145 from wild boar in France, Germany, Austria and Hungary, all of which were sequenced in the frame of a large study investigating *S.* Choleraesuis var. Kunzendorf in European pigs and wild boar [25].

**Figure 3:**
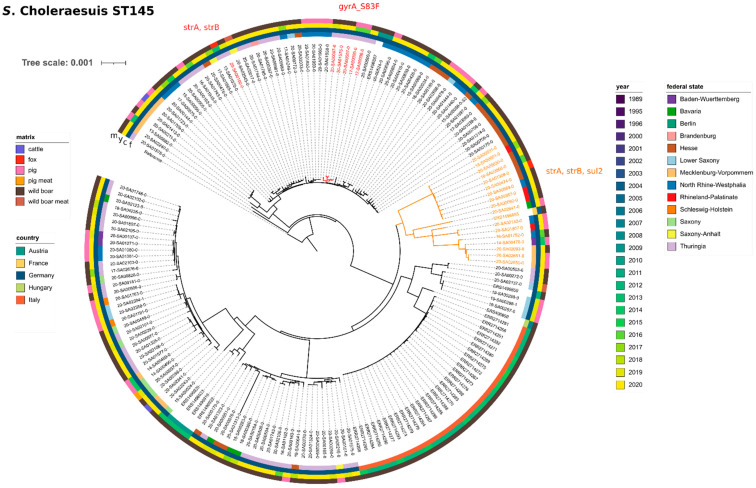
Phylogenetic relationship between *S.* Choleraesuis (ST145) wildlife isolates and isolates from food, feed and farm animals. Maximum likelihood phylogenetic tree based on core SNPs in the bacterial chromosome. The chromosome sequence of *S.* Choleraesuis str. SCSA50 isolate A50 (NZ_CM001062.1) was used as reference. The tree was computed with the snippySnake pipeline with IQ-TREE, visualized in iTOL and manually rooted to the reference. The colour bars (inside to outside) identify the German federal state (f), national country (c), sampling year (y) and matrix (m) of the samples. Isolates carrying antimicrobial resistance genes are highlighted in colour with the type of resistance genes indicated. The scale bar shows the genomic distances of the sequences in substitutions per site.

In summary, we compared a set of 177 isolates (wildlife dataset *n* = 96, in-house data from farm animals and food *n* = 43, external wildlife data *n* = 38) by SNP calling. The analysis yielded a median of 236 SNP differences among these isolates (Supplementary File S5). The SNP-based maximum-likelihood phylogenetic tree (Figure 3) shows that isolates from wild boar, red foxes, domestic pigs and food are randomly interspersed throughout the phylogenetic tree. Isolates clustered according to countries of origin, with the Italian wild boar isolates (from 2012–2015) forming the most distinct cluster. Interestingly, the most closely related isolate to the Italian cluster did not originate from a neighbouring country, but from Hungary (from 2007). The oldest isolate in the dataset was obtained in 2004 from a wild boar in France (ERS1496855), which clustered with recent isolates from German pigs and wild boar from 2016–2020. Overall, although there is some clustering according to the year of isolation, geographical origin (on country level) appears to correlate better with clustering results than the isolation date. On a regional level, the German isolates did not show a strong clustering behaviour according to the federal state.

The majority of investigated wildlife isolates of ST145 (including the external sequence data from wild boar in Italy, France and Hungary) did not possess AMR genes. However, eleven isolates (fox *n* = 1 and wild boar *n* = 10) featured a set of three mobile AMR genes, namely *aph(3*′′*)-Ib* (*strA*)*, aph(6)-Id* (*strB*) and *sul2*, conferring resistance against the aminoglycoside streptomycin and sulfonamide antibiotics (sulfamethoxazole), respectively. All three genes were predicted to be located on a small plasmid of ~8800 bp size with plasmid marker IncQ1_1 (closed plasmid sequences available for 16-SA01752-0, 16-SA02866-0 and 18-SA00001-0). In another isolate (20-SA00100-0), only the genes *aph(3*′′*)-Ib* (*strA*) and *aph(6)-Id* (*strB*) could be detected, also located on a plasmid contig (plasmid marker IncI1_1_Alpha). Sulfamethoxazole resistance was confirmed experimentally for one isolate (20-SA00387-0) (Supplementary File S1). In the phylogenetic tree, all isolates carrying the full set of the three AMR genes formed one cluster, together with one isolate from a domestic pig and the historic isolate from France. In addition, three isolates from the German state of Thuringia (all 2020) featured point mutations in the chromosomally located *gyrA* gene (*gyrA_S83F*)*,* conferring resistance against (fluoro-)quinolone antibiotics (nalidixic acid/ciprofloxacin). Resistance against nalidixic acid and ciprofloxacin was confirmed experimentally in one isolate (20-SA01576-0) (Supplementary File S1). In the phylogenetic tree, these isolates form one cluster, together with two isolates from domestic pigs.

### 3.4. Detailed Analyses and Phylogenetic Study of Serovar S. Enteritidis, ST11 in Wildlife, Especially Foxes

*S.* Enteritidis, ST11, was the most frequent serovar isolated from foxes. Of the 44 isolates obtained from foxes, 26 isolates were serotyped as serovar *S.* Enteritidis (59.1%). All but two of these isolates were of ST11 (the other two were ST183). For one isolate, no ST could be determined. In addition to foxes (*n* = 23), *S.* Enteritidis ST11 was also detected in other wildlife (*n* = 22). However, compared to the total number of investigated isolates, foxes had the highest prevalence of *S.* Enteritidis ST11 (compare: wild boar (5/112, 4.5%), deer (4/12, 33.3%), raccoon (3/12, 25%), pigeon (2/19, 10.5%), wild birds (7/37, 18.9%)). For a phylogenetic analysis, we included short-read data of *S.* Enteritidis ST11 from our in-house database of domestic animals and food isolates. As this database holds a large number of *S.* Enteritidis ST11 isolates (*n* ≥ 600) (mostly from chicken, eggs and chicken meat), we applied the R function *sample_n* to randomly select 70 isolates. For comparison with international data, we included short-read data of two isolates with ST11 obtained from wild chicken in the US between 1995 and 1996 (SRR5387492, SRR5387496) [26].

**Figure 4:**
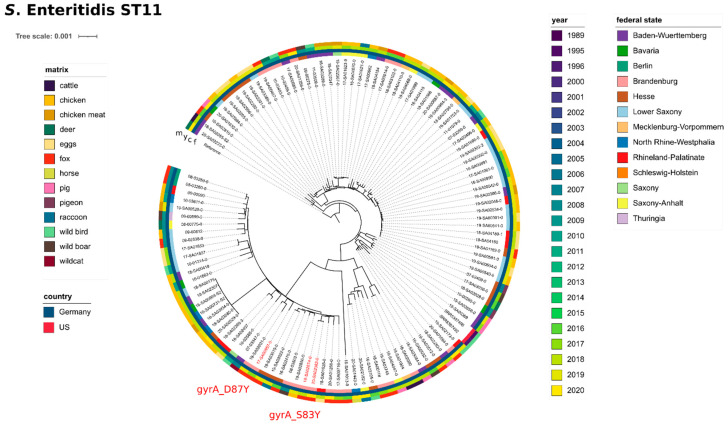
Phylogenetic relationship between *S.* Enteritidis (ST11) wildlife isolates and isolates from food, feed and farm animals. Maximum likelihood phylogenetic tree based on core SNPs in the bacterial chromosome. The chromosome sequence of *S.* Enteritidis strain ATCC BAA-708 (NZ_CP025554.1) was used as reference. The tree was computed with the snippySnake pipeline with IQ-TREE, visualized in iTOL and manually rooted to the reference. The colour bars (inside to outside) identify the German federal state (f), national country (c), sampling year (y) and matrix (m) of the samples. Isolates carrying antimicrobial resistance genes are highlighted in colour with the type of resistance genes indicated. The scale bar shows the genomic distances of the sequences in substitutions per site.

In total, we compared a set of 117 *S.* Enteritidis ST11 isolates (wildlife dataset *n* = 45, in-house data from domestic animals and food *n* = 70, external wildlife data *n* = 2) by SNP calling. Overall, we detected a high genetic diversity with a median of 330 SNPs among these isolates (Supplementary File S5). The SNP-based maximum-likelihood phylogenetic tree (Figure 4) shows that the sampling year does not correlate well with the clustering results, with historic isolates clustering closely with current isolates. Regional geography (German federal states) does not appear to affect clustering. The matrices also do not have a strong influence on clustering results, as seen by the fact that fox isolates, as well as other wildlife isolates, do not form separate clusters, but are randomly distributed throughout. The majority of ST11 isolates did not harbour any mobile AMR genes or AMR-related point mutations. Three isolates (all obtained from foxes in Brandenburg, 2017–2020) featured point mutations in the *gyrA* gene (either *gyrA_D87Y* or *gyrA_S83Y*), conferring resistance against fluoroquinolone antibiotics (nalidixic acid/ciprofloxacin).

### 3.5. Detailed Analyses and Phylogenetic Study of Serovar S. Enteritidis, ST183 in Hedgehogs

**Figure 5:**
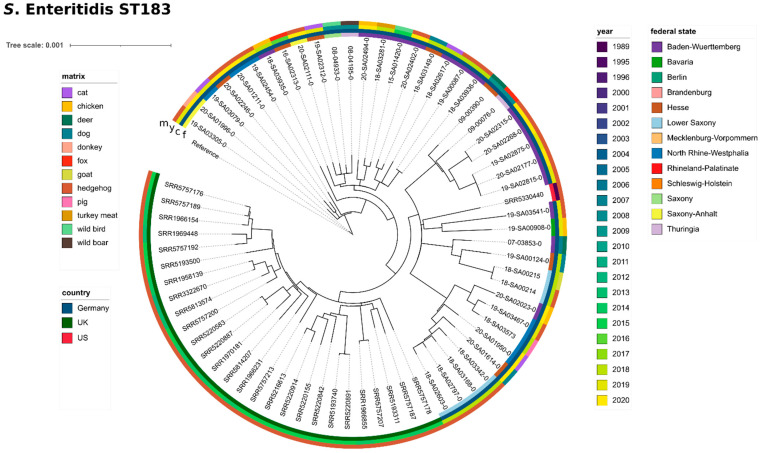
Phylogenetic relationship between *S.* Enteritidis (ST183) wildlife isolates and isolates from food, feed and farm animals. Maximum likelihood phylogenetic tree based on core SNPs in the bacterial chromosome. For lack of a publicly available complete genome sequence for *S.* Enteritidis ST183, a draft assembly of 19-SA03305-0 was used as a reference. The tree was computed with the snippySnake pipeline with IQ-TREE, visualized in iTOL and manually rooted to the reference. The colour bars (inside to outside) identify the German federal state (f), national country (c), sampling year (y) and matrix (m) of the samples. Isolates carrying antimicrobial resistance genes are highlighted in colour with the type of resistance genes indicated. The scale bar shows the genomic distances of the sequences in substitutions per site.

*S.* Enteritidis, ST183, was the only serovar isolated from hedgehogs (*n* = 20). In addition, we detected ST183 in seven other wildlife isolates (fox *n* = 2, deer *n* = 2, wild birds *n* = 2, wild boar *n* = 1). For a phylogenetic analysis, we included all remaining ST183 isolates from our in-house database, consisting of isolates from pet animals (cat *n* = 4, dog *n* = 3), farm animals (chicken *n* = 4, goat *n* = 1, pig *n* = 1, donkey *n* = 1) and one food isolate (turkey meat). For comparison with international data, we included short-read data of 48 isolates with ST183 (phage type: PT11 *n* = 27, PT66 *n* = 21), sequenced in the frame of a large study investigating ST183 in hedgehogs and people in Britain [6], as well as one isolate with ST183 from a hedgehog in the U.S. obtained in 1989 (SRR5330440) [26]. When comparing our data to the UK isolates, we found that our isolates clustered with the UK isolates of phage type (PT) 11, thereby suggesting that our isolates belong to PT11 (data not shown). In addition, three of the isolates were previously experimentally confirmed as PT11 (Supplementary File S1). Since 2012, phage typing is no longer implemented routinely at the German NRL-*Salmonella;* however, former PT results revealed that from 79 *S.* Enteritidis isolates obtained from hedgehogs, 31 belonged to PT11 (others: one PT21, one PT4, 22 did not conform to a designated type (RDNC) and 24 were not tested). For greater resolution, we chose to include UK PT11 isolates only in the subsequent phylogenetic analysis. In total, we compared a set of 70 isolates (wildlife dataset *n* = 27, in-house data from domestic animals and food *n* = 15, external wildlife data *n* = 28) by SNP calling. Overall, we detected a low genetic diversity with a median of 95 SNPs among these isolates (Supplementary File S5). The SNP-based maximum-likelihood phylogenetic tree (Figure 5) shows that isolates strongly cluster according to country of origin. Interestingly, the single U.S. isolate from 1998 clusters within the German isolates. Regional geography (German federal states) does not correlate well with the clustering results. Similarly, the isolation year does not seem to affect clustering results, with isolates obtained ten years ago clustering closely with current isolates. Isolates from domestic animals, as well as from other wildlife animals do not form separate clusters, but are randomly distributed throughout the hedgehog isolates. No AMR genes were detected in any of the ST183 isolates, which was additionally confirmed by MIC testing of four isolates, which were susceptible to all antimicrobials tested.

A plasmid of size ~87,300 bp (plasmid marker IncFII(S)_1) was predicted in all wildlife *S.* Enteritidis ST183 isolates (closed plasmid sequences available for 07-03853-0, 18-SA00214, 18-SA02603-0, 18-SA03342-0, 19-SA02815-0, 19-SA02875-0, 19-SA03467-0 and 19-SA03541-0).

### 3.6. Occurrence of S. Typhimurium, ST128, in Pigeons

*S.* Typhimurium, ST128, was the dominant serovar isolated from pigeons. Of the 19 isolates obtained from pigeon, 16 isolates were serotyped as serovar *S.* Typhimurium (84.2%). All but two of these isolates belong to ST128 (the remaining were ST19). For one isolate, no ST could be determined (due to a missing allele). In addition, we detected ST128 for one isolate obtained from deer (07-04265-0). In our in-house collection of sequenced *Salmonella* spp. from farm animals and food, we found only two other isolates, both obtained from laying hens, with ST128 (19-SA00476 and 19-SA02615), which we included for comparison in the phylogenetic analysis. SNP analysis yielded a median of 109 SNPs between these sixteen isolates, with no notable differences among the matrices (Supplementary File S5). No AMR genes were detected in the majority of the isolates. However, isolate 20-SA01154-0 (pigeon, 2020) featured a plasmid contig (plasmid marker IncI1_1_Alpha), with three mobile AMR genes *bla*_TEM-1_, *dfrA1* and *sul2*, conferring resistance against several beta-lactamase (e.g., ampicillin), trimethoprim and sulfonamide antibiotics (sulfamethoxazole) respectively.

### 3.7. Occurrence of Serovar S. Ball, ST3502, in Raccoon

Our dataset contains twelve isolates from raccoon, collected between 2017 and 2020. Half of the raccoon isolates were serotyped as serovar *S.* Ball (seroformula (1),4,5,12:y:e,n,x), ST3502. All six were collected in the first half of 2020 and from a very narrow geographic region (Saxony-Anhalt *n* = 5 and Brandenburg *n* = 1). These isolates were collected in the frame of an investigation into a serovar *S.* Ball occurrence in the region [7]. In addition, serovar *S.* Ball ST3502 was also detected in one wild boar from 2019. SNP analysis yielded an average of 12 SNPs between the seven wildlife isolates (Supplementary File S5). No AMR genes or plasmid sequences were detected in any of the *S.* Ball isolates.

### 3.8. Salmonella in Rare Predators (Wolf and Wild Cat)

Our data set contained *Salmonella* spp. from three rare predators (two from wolf, one from wildcat). Wolves have been returning to Germany in the past 20 years, after becoming extinct in 1850. The two *Salmonella* isolates from wolves were obtained from a wolf from Brandenburg in 2008 (08-02443-0) and from the small intestine of a wolf from North Rhine-Westphalia in 2019 (19-SA01791-0). The older isolate was found to be serovar *S.* Typhimurium ST19, and the more recent isolate was serotyped as *S.* Typhimurium monophasic (I 4,[5],12:i:-) ST34. While the older isolate possessed no AMR genes and carried no plasmid, the 2019 isolate featured a considerable number of AMR genes. The isolate was found to carry three mobile AMR genes, namely *aph(3*′′*)-Ib* (*strA*)*, aph(6)-Id* (*strB*) and *sul2*, conferring resistance against the aminoglycoside streptomycin and sulfonamide antibiotics (sulfamethoxazole), respectively. In addition, the isolate possessed the *bla*_TEM-1_ (beta-lactamase) gene, which could not be assigned to any plasmid contig, as well as the *tet(B)* (tetracycline) gene (both possible chromosomally located).

Wildcats are another smaller, predator species. An *S.* Enteritidis isolate (ST11) was obtained in 2007 from a European wildcat in Baden-Württemberg (07-03847-0). No AMR genes were detected in the sequence data of this isolate.

### 3.9. Wildlife–Human Transmissions of Serovars S. Enteritidis, S. Choleraesuis and S. Typhimurium

In order to identify wildlife–human transmission events for the most common serovars, especially *S.* Enteritidis and *S.* Typhimurium as category 1 *Salmonella* with highest public health significance [27], and to explore potential links to farm and production environments, we performed a joint cgMLST calling of the wildlife isolates together with human, farm and food isolates.

For this analysis, we included 1864 isolates from farm and food from our in-house collection (NRL-*Salmonella* database) and 1046 human isolates collected in 2020 in the frame of the ongoing GenoSalmSurv real-time surveillance project [4], plus an additional 71 human isolates included in recent outbreak investigations. An overview of the included isolates grouped by serovar and database is given in Table 2. We aimed to detect clusters of close wildlife–human and wildlife–non-human matches, with ≤5 allele differences for *S.* Choleraesuis and *S.* Typhimurium (both mono- and biphasic) and ≤3 ADs for *S.* Enteritidis, containing at least one human isolate. Other serovars were not considered. After identifying suitable clusters with cgMLST, we performed SNP calling for selected clusters and estimated maximum-likelihood-based phylogenetic trees from the core SNPs. cgMLST/SNP distance matrices for selected clusters, as well as associated metadata are listed in Supplementary File S6. For easier referencing, clusters are identified by the respective names attributed to them in the GenoSalmSurv Project.

**Figure 6:**
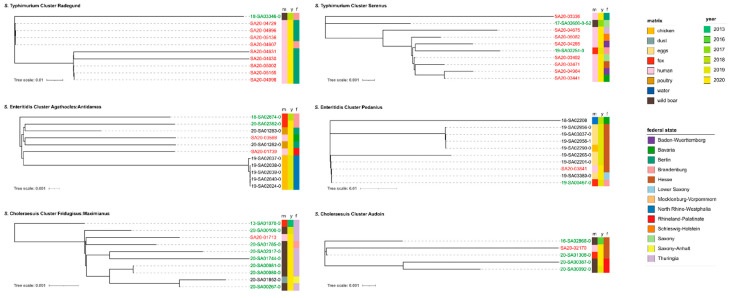
Phylogenetic trees of mixed-matrices clusters for the serovars *S.* Typhimurium (ST19), *S.* Enteritidis (ST11) and *S.* Choleraesuis (ST145). Maximum-likelihood phylogenetic tree based on core SNPs in the bacterial chromosome. The chromosome sequences of *S.* Typhimurium strain 16A242 (NZ_CP020922.1), *S.* Enteritidis strain ATCC BAA-708 (NZ_CP025554.1) and *S.* Choleraesuis strain SCSA50 isolate A50 (NZ_CM001062.1) were used as references. The trees were computed with the snippySnake pipeline with IQ-TREE, visualized in iTOL and manually rooted to the reference. The colour bars (left to right) identify the matrix (m), sampling year (y) and German federal state (f) from which the samples were obtained. Human clinical isolates are highlighted in red; wildlife isolates are highlighted in green. The scale bars show the genomic distances of the sequences in substitutions per site.

For *S.* Typhimurium we found two large clusters (both ST19). In the first cluster (cluster name: Radegund.Alpha), one wild boar sample from 2018 clustered with nine human clinical isolates from 2020 with 2–4 ADs (4–8 SNPs, respectively). All isolates were collected from the same geographic region (Berlin/Brandenburg). ADs within the human isolates were smaller (mean AD: 1.2/mean SNP difference: 2.3), than between human and wildlife isolates (mean AD: 2.9/mean SNP difference: 5.2), probably due to the narrow time span in which the human isolates were collected (over the course of 25 days in September 2020). This is also reflected in the phylogenetic tree (Figure 6), which shows the wild boar sample separated from the human isolates.

In the second *S.* Typhimurium cluster (cluster name: Serenus.Delta), two wildlife samples clustered with 10 human clinical isolates from 2020 with 1–6 ADs. One wildlife sample was obtained from wild boar in 2017 in Saxony. The other was obtained from a fox in 2019 in Brandenburg (19-SA02251-0). The human isolates were collected over the course of three months from early July 2020 to late September 2020. Interestingly, the isolates originated from a wide geographic region (8 different federal states). Again, the ADs within the human isolates were smaller (mean AD: 1.9/mean SNP difference: 5.9), than between human and wildlife isolates (mean AD: 3.3/mean SNP difference: 6.9), although the wildlife isolates did not cluster separately from the human isolates in the phylogenetic tree (Figure 6).

For *S.* Enteritidis we found four large clusters (all ST11), involving human isolates, wildlife isolates (mostly from foxes), food isolates (mainly eggs) and farm isolates (mainly chicken). We decided to explore two of these clusters in more detail. In the first cluster (cluster name: Agathocles:Antidamas.Delta) two fox isolates from Brandenburg, obtained in 2018–2020, clustered with two human clinical isolates, as well as five chicken and two poultry meat isolates with 3–4 ADs (3–5 SNPs respectively). The five chicken isolates originated from one laying-hen farm in North Rhine-Westphalia and were isolated on the same day, which is reflected in the phylogenetic tree (Figure 6) and 0 SNPs difference between these isolates. In contrast, the two poultry isolates (obtained from the same production site) clustered more closely with the human isolates, as well as with the fox isolate from 2020. The older fox isolate showed a greater distance in the phylogenetic tree. Overall, all samples clustered very closely with a mean AD of 2.4 and a mean SNP difference of 3.6.

In the second *S.* Enteritidis cluster (cluster name: Pedanius.Alpha) another fox isolate from Brandenburg clustered with one human isolate, as well as six food isolates (eggs), one farm isolate (chicken) and one environmental isolate (water) with 1–3 ADs (0–2 SNPs respectively). All isolates were obtained between 2018 and 2020. The majority of egg isolates originated from Hesse, with one egg isolate from Lower Saxony. In the phylogenetic tree, the chicken isolate (also from Hesse) clusters closely with three of the five egg isolates from Hesse. The human isolate was obtained in 2020 from a patient also in Hesse and clusters together with the fox isolate, the egg isolate from Lower Saxonyand another egg isolate from Hesse. The oldest isolate in the cluster, obtained from an environmental water sample from Bavaria, is shown most distantly related to all other isolates in the phylogenetic tree. Overall, all isolates cluster very closely with a mean AD of 1.2 and a mean SNP difference of 1.1.

For *S.* Choleraesuis we detected three larger clusters, all of which predominantly involved wild boar isolates. We investigated two of these clusters in more detail (all ST145). In the first cluster (cluster name: Fridugisus:Maximianus), eight wildlife isolates (seven wild boar, one fox) clustered with one human isolate and one isolate obtained from the surroundings of a feed mill. Wildlife isolates, as well as the human isolate, were collected from the same geographic region (Thuringia/Brandenburg) during the years 2019–2020. Interestingly, the fox isolate was obtained in 2013, which is also evident from the phylogenetic tree (Figure 6), which shows this isolate further distant from isolates sampled more recently. The farm isolate was obtained in 2020 from a farm in Saxony-Anhalt. Neither the farm isolate nor the human isolate clustered separately from the wildlife isolates. Overall, all isolates clustered with a mean AD of 4.1 and a mean SNP difference of 7.7 SNPs.

In the second, smaller *S.* Choleraesuis cluster (cluster name: Audoin), four wildlife isolates (three wild boar, one fox) clustered with one human isolate. All five isolates were collected from the same geographic region (Rhineland-Palatinate/Hesse) during the years 2016–2020. Again, the phylogenetic tree (Figure 6) shows the oldest isolate separate from the later isolates. The human isolate clustered most closely with the fox isolate. Overall, all samples clustered with a mean AD of 2.9 and a mean SNP difference of 3.4 SNPs.

## 4. Discussion

### 4.1. Wildlife Isolates Have An Overall Low Prevalence of AMR Genes, with Increased Occurrence in Wild Birds

We found that the majority of investigated isolates was pan-susceptible (85.8%), and that only a small set of 37 isolates carried AMR genes. Thus, wildlife isolates appear to have an overall lower burden of AMR, as concluded in other studies [19,28,29]. Screening our own in-house database of *Salmonella* isolates from farm animals and foodstuff in Germany for comparison (which comprises a representative selection of serovars from the German federal states and food-production sectors), we found that more than half of those isolates possessed one or more AMR genes (data not shown). Therefore, AMR carriage in wildlife (14.2%) is much lower than in isolates from production environments. The observed types of AMR genes and phenotypic resistances in the wildlife isolates mirrored those commonly found in *Salmonella* isolates from human, farm animals and foodstuff [30,31,32,33]. A small number of isolates carried resistance to important WATCH-group antibiotics, such as fluoroquinolones or macrolides. Although overall prevalence was low, AMR gene carriage was slightly higher in wild birds, which were also more likely to exhibit phenotypic resistances against two or more classes of antimicrobials. Almost one-quarter of all wild bird isolates, as well as two pigeon isolates, were found to possess AMR genes. Curiously, AMR-positive isolates originated from very different types of wild birds, including songbirds (starling), water birds (mallard, mute swan), birds of prey (owl, buzzard), game birds (quail), crows (raven, rook) and migratory birds (stork). Therefore, no common link regarding habitat, feeding behaviour or social interactions can be inferred. However, as described for an NDM-1 carbapenemase-producing *S.* Corvallis isolate from a black kite in Germany in 2013 [34,35] and other studies on bacterial isolates from migratory birds and water birds [36,37], these bird species were shown more prone to AMR carriage. Proximity to human settlements was also found to be a contributing factor [38,39]. As Germany is among the most densely populated countries in Europe, most wild birds live close to human environments and activities. Thus, any wild bird species may be exposed to antibiotic-resistant bacteria found at livestock farms, landfills or waste-water treatment facilities.

### 4.2. The Swine-Adapted S. Choleraesuis var. Kunzendorf, ST145 Shows High Prevalence in Wild Boar and Occasionally Carries Multidrug Resistance

*S.* Choleraesuis var. Kunzendorf is a serovar adapted to swine that can also cause infections in humans [40]. Human-associated outbreaks caused by *S.* Choleraesuis var. Kunzendorf are rare, and no major outbreak has been detected in Germany in recent years. In Germany, *S.* Choleraesuis var. Kunzendorf is not among the dominant serovars detected in domestic pigs [4,41], accounting for less than 1% of detected *Salmonella enterica* serovars in pigs. In contrast, *S.* Choleraesuis var. Kunzendorf is the dominant serovar in wild boar in Germany (85.7%). An older study found *S.* Choleraesuis to be the only *Salmonella* serovar detected in wild boar from the German state of Thuringia [41]. The majority of *S.* Choleraesuis isolates were identified as ST145 (97.9%). This finding confirms that wild boar act as a wildlife reservoir for this sequence type [24,25]. The majority of isolates investigated in this study were found to be pan-susceptible, and therefore initial colonization of wild boar with this serovar may have occurred decades ago, as speculated by Leekitcharoenphon et al. (2019) [25], which is further supported by other studies [24]. In addition to wild boar, our study also contains two *S.* Choleraesuis isolates from foxes. Presence in foxes, badgers and deer in Germany has been reported previously [42]. Inferring from a phylogenetic analysis, including data from previous international studies, we found that wild boar isolates clustered according to geographic origin on a national level. However, on a regional level no strong clustering tendencies were apparent. We suspect that colonization of wild boar with *S.* Choleraesuis has occurred sufficiently long ago that individual variants now circulate broadly in the German wild boar population. We further found isolates from wild boar, red foxes, domestic pigs and foodstuff randomly interspersed throughout the phylogenetic tree, indicating frequent transmission events. Inter- and intra-species transmission may be facilitated by the fact that both wild boar and foxes frequent urban and rural areas and exhibit scavenging behaviour (including the eating of dead animals).

Although the majority of isolates were pan-susceptible (84.4%), we detected mobile AMR genes (*aph(3*′′*)-Ib* (*strA*)*, aph(6)-Id* (*strB*)), *sul2* (located on small IncQ1_1 plasmids) and point mutations in the *gyrA* gene (*gyrA_S83F*) in 15 wild boar and red fox isolates. Isolates with the same resistance profile have been reported previously in Danish and French pig [43], and wild boar from France, Germany and Italy [25,41,44]. From our analysis, we can conclude that this multidrug-resistant variant has been in circulation since at least 2004 and that it is also found sporadically in livestock. To our knowledge, the described point mutations in the *gyrA* gene have not been previously observed in European wild boar, although one study reported two nalidixic-acid-resistant strains (from 2011 and 2014), derived from wild boar kept on Italian game estates [44]. Fluoroquinolone-resistant *S.* Choleraesuis strains were first described around 1998–2004 in Taiwan [45] and have also been detected in Japan [46]. The same base substitution at codon 83 (TCC→TTC), as reported by Chiu et al. (2002) [45] was also observed in this study. We further detected a base substitution at codon 57 (ACC→AGC) of the *parC* gene that likely contributes to the resistance phenotype. It has been speculated that this resistant variant arose due to the use of antibiotics added to animal feed as a growth promoter and has subsequently spread into wildlife [45].

### 4.3. The Chicken-Associated Serovar S. Enteritidis ST11 Is Also Common in Wildlife, Especially in Foxes, without Genetic Divergence

The serovar *S.* Enteritidis is a common cause of human salmonellosis worldwide, with a high prevalence in livestock and food. In Germany, *S.* Enteritidis is the most prevalent serovar in laying hens and is also frequently detected in broilers [4]. Generally, the most common sequence type of *S.* Enteritidis is ST11 (ST11 is also the most frequent ST on the Enterobase platform (18% of 286,505 *Salmonella* spp. strains, as of 9 February 2021)).

In this study, we detected *S.* Enteritidis ST11 in various wildlife species (fox, wild boar, deer, raccoon, pigeon, wild birds, wildcat), with an especially high prevalence in foxes (23/44, 52%). In contrast, other studies have found *S.* Typhimurium to be the dominant serovar in foxes in Italy [47], Norway [48] and Poland [49]. In Austria, serovar *S.* Dublin was found to be the dominant serovar in foxes [50]. Infrequently, *S.* Enteritidis was also detected in foxes in Italy [47] and Spain [51].

When screening our in-house database of *Salmonella* spp. isolates from domestic animals and food for *S.* Enteritidis, we found the majority of *S.* Enteritidis isolates originating from chicken, eggs and chicken meat. With a few exceptions, all could be attributed to ST11.

A phylogenetic comparison between the wildlife ST11 isolates, as well as random selection of food and farm isolates showed that wildlife isolates do not form a separate cluster (or cluster by species), but are randomly distributed within the isolates from domestic animals and foodstuff, thus indicating frequent transmission events. It is unclear whether the increased prevalence of ST11 in foxes reflects a direct link between foxes and chickens (which are commonly associated with ST11) or is an incidental finding. One possibility is that foxes may seek out chicken farms (especially those with large outdoor facilities producing free-range eggs) in search of food, or encounter discarded chicken meat, eggshells or egg products when foraging in cities or settlements. As foxes frequent both urban and rural environments, they may then transmit the bacteria to other wildlife species. Foxes may also indirectly come in contact with ST11 by predating on small rodents or wild birds feeding of spilled grains/surplus chicken feed or insects on such farms.

Overall, we detected a high genetic diversity (median of 330 SNPs), reflecting the diverse nature of this sequence type. Neither regional geography nor isolation date showed strong correlation with the clustering results. Although the majority of isolates were pan-susceptible (93.3%), we found three isolates with point mutations in the *gyrA* gene, conferring resistance against fluoroquinolone antibiotics.

### 4.4. The Hedgehog-Associated Serovar S. Enteritidis, ST183, Was the Sole Serovar Isolated from Hedgehogs

*S.* Enteritidis ST183 has been found associated with hedgehogs in Germany [52], Great Britain [6,53,54] and Denmark [55]. A large study found that *S.* Enteritidis ST183 was the only *Salmonella* serovar isolated from 46 UK hedgehogs, with both PT11 and PT66 detected [6]. Equally we characterized all our hedgehog isolates (*n* = 20) as *S.* Enteritidis ST183 (PT11). Additionally, we detected ST183 in seven other wildlife isolates (fox, deer, wild birds, wild boar), as well as domestic animals (pig, chicken, donkey, goat) and pet animals (cat, dog). Sporadic incidence in livestock and companion animals was also observed in the UK [55] and Germany [56]. The observation that *S.* Enteritidis ST183 is occasionally detected in pet animals is likely linked to the fact that hedgehogs, cats and dogs share the same residential areas (gardens, parks, meadows and other green spaces). In addition, hedgehogs are often fed by garden owners and may feed from the same bowls as pet animals. In our study, isolates did not cluster by host species, indicating frequent inter-species transmission events. Although the included set of UK isolates clustered separately from the German isolates, we observed a relatively low overall genetic diversity (median of 95 SNPs). This was confirmed by a historic U.S. isolate of 1998 with close phylogenetic similarity to current German isolates. None of the tested ST183 isolates exhibited phenotypic antibiotic resistance, and no genotypic resistance determinants were detected. This is in line with Lawson et al. (2018) [6], who found all 46 hedgehog isolates investigated in this study susceptible to antibiotics. The absence of AMR for this sequence type suggests a relative restriction to wildlife habitats, with little exposure to places of high antibiotic use, such a livestock farms or human clinical settings.

However, despite the fact that *S.* Enteritidis ST183 seems to be especially related to hedgehogs, ST183 is not strictly host-specific, as it has been found in a broad range of other species, including humans. A similar conclusion was drawn by Sangal et al. (2009) [57], who described *S.* Enteritidis ST183 as host-adapted, but not strictly host-restricted to hedgehogs. Our results confirm the observation that the sequence type is host-adapted to hedgehogs and that hedgehogs are the principle reservoir of ST183.

### 4.5. The Pigeon-Adapted S. Typhimurium ST128 Is the Dominant Sequence Type in Pigeons

*S.* Typhimurium ST128 (also defined by phage typing as DT2) is a single-locus variant of the common sequence type ST19 and has previously been reported to be host-restricted to pigeons [58,59]. Indeed, the majority of pigeon isolates (13/19, 68.4%) in our data set were typed as Typhimurium, ST128 (DT2). We observed a relatively low overall genetic diversity (median of 109 SNPs). Although the great majority of our wildlife ST128 isolates were pan-susceptible (14/15, 93.3%), we predicted a plasmid featuring three mobile AMR genes (*bla*_TEM-1_*, dfrA1 and sul2*) in one pigeon isolate from 2020. The occurrence of *bla*_TEM-1_ and *sul1*/*sul2* has been observed previously in *S.* Typhimurium isolates from pigeons in Poland [60] and Egypt [61].

### 4.6. The Detection of Serovar S. Ball ST3502 in Raccoon as an Incidental Finding and Its Potential to Emerge as a New Persisting Serovar in Germany

Serovar *S.* Ball is a rare and little-investigated serovar that first appeared as a food-borne pathogen in RASFF notifications from 2014 (in mushrooms and spices from Hungary, Poland and Vietnam). In Germany, *S.* Ball was only sporadically detected before 2019, but the number of *S.* Ball isolates received by the NRL-*Salmonella* has increased significantly in the past two years, with isolates mainly originating from wildlife animals (raccoon, wild boar) and from the beef production chain (calves, cattle). Although half of our raccoon isolates were serotyped as serovar *S.* Ball, we believe this is not necessarily linked to an increasing prevalence of this serovar in German raccoon. On one hand it reflects a sampling strategy linked to a targeted examination of *S.* Ball occurrence in German livestock farms and surroundings. On the other hand, it reveals that raccoon can act as a reservoir of this serovar. Our phylogenetic analysis showed a very close genetic relationship between these isolates with an average of 7 SNP differences. These results may indicate a clonal distribution of this serovar due to one single importing event into German livestock and environment or wildlife. Therefore, the detection of serovar *S.* Ball in raccoon is likely an incidental finding, linked to a specific occurrence of *S.* Ball in the region or its raccoon population. This is further supported by the fact that of the 55 isolates obtained from raccoon listed in the NRL collection (not sequenced), none has previously been serotyped as serovar *S.* Ball. Instead, the majority were serotyped as *S.* Typhimurium (21/55, 38%), followed by *S.* Stourbridge (7/55, 13%) and *S.* Wangata (5/55, 9%) (data not shown). In this study, the remaining six raccoon isolates were serotyped as *S.* Enteritidis (*n* = 4), Coeln (*n* = 2) and *S.* Typhimurium (*n* = 1). Another study detected the serovars *S.* Typhimurium and *S.* Newport in raccoon in Poland [49]. However, since serovar *S.* Ball was also recently detected in the livestock sector in Germany and is also increasingly detected in humans in Germany [7], occurrence of *S.* Ball in Germany, whether in raccoon or other species, deserves further attention in order to monitor whether this recently emerging serovar establishes itself as a new persisting serovar.

### 4.7. Transmission of Salmonella spp. between Human and Wildlife

The relationship between *Salmonella* in wildlife and human infections is still poorly understood, although recent outbreak investigations have detected outbreak strains in wildlife animals and surroundings. We therefore attempted to analyse potential wildlife–human links present in our data set. In order to identify close clusters between wildlife and human isolates, we performed cgMLST and SNP calling on wildlife isolates, together with human isolates from recent surveillance and outbreak studies. We further included isolates from farm animals and food to detect potential links between wildlife, farm environments, food production and human health. We identified several mixed-matrices clusters for *S.* Typhimurium, *S.* Enteritidis and *S.* Choleraesuis. We found that the composition of these clusters differentiated by serovar. For example, for *S.* Typhimurium, the two analysed clusters contained predominantly human samples, which were linked to one or more wildlife samples. In contrast, clusters for *S.* Choleraesuis contained mostly wildlife samples with only one human sample each. For *S.* Enteritidis, food and farm isolates (mainly chicken and eggs) prevailed in both clusters. In all cases, wildlife isolates originated from either wild boar or foxes. Based on this limited data we speculate about the causal relationship and transmission events that may underlie these clusters.

For *S.* Typhimurium, we assume that for both clusters the origin is a persistent strain in a farm environment, as *S.* Typhimurium ST19 is predominantly associated with farm animals. In our study, *S.* Typhimurium ST19 was not a serovar typically detected in wild boar or foxes, and therefore a transmission chain from farm to wildlife is assumed. Wildlife animals may be exposed through scavenging of food waste (both wild boar and foxes are scavenging species that frequent urban environments), direct contact with the farm environment or indicted contact through wastewater/sewage from the farm or infected humans. Human infections are likely caused by the consumption of contaminated food. Unfortunately, neither of the two clusters contained a farm or food isolate, which may pinpoint the type of farm environment. However, as *S.* Typhimurium is the dominant serovar in pigs and cattle [4], either contaminated pork or beef seems a likely cause of the human infections. A nationwide distributed product could also explain the wide geographic distribution of the human isolates observed in the cluster.

For *S.* Enteritidis, the overall picture is clearer. We previously found that ST11 is the main ST observed in poultry, as well as foxes. As both investigated clusters predominantly contained chicken, other poultry and egg isolates, the origin of these clusters can be pointed to a persistent strain in a farm environment (likely chicken or poultry). The link to the involved fox isolates has been discussed earlier. Briefly, a transmission from farm to wildlife through scavenging behavior or direct contact of foxes with the farm environment is the most plausible transmission chain. Again, human infections can be explained through the consumption of contaminated food products (either chicken meat or eggs).

For *S.* Choleraesuis, contrary to *S.* Typhimurium and *S.* Enteritidis, we do not assume a farm-associated strain as the starting point. As we and others have shown, *S.* Choleraesuis is a pig-adapted serovar with wild boar as the main reservoir in Europe. Combined with the fact that in both clusters wildlife isolates (mostly wild boar) prevail, we assume a wildlife origin. Transmission of *S.* Choleraesuis from wild boar to humans could occur through the consumption of wild boar meat or indirectly, for example, through consumption of contaminated grains, fruit or vegetables either grown on commercial fields or private gardens, which the omnivorous wild boar seek out for food. Very rarely, Choleraesuis is also detected in domestic pigs, so that a transmission from domestic pigs or pork to humans cannot be ruled out. Transmission from wild boar to domestic pigs could, for example, occur through contamination of feed (corn, soya, silage) or bedding material (hay, grass, straw). However, as pig farms usually have a very high biosecurity level, further enhanced by the threat of African Swine Fever [62], a transmission from wild boar to domestic pigs is unlikely (with the exception of free-range pigs). A similar conclusion was drawn by Longo et al. (2019) [24], who found that a set of 30 wild boar isolates was phylogenetically related to one human isolate from the same area with an average of 45 SNPs difference.

Overall, we believe that the majority of *Salmonella* spp. transmission events follows the direction farm to wildlife (and farm to human), as opposed to wildlife to farm (or wildlife to human), as there are a considerably greater number of food-producing animals than wild animals, with a subsequent negative impact on natural environments. This is clearly demonstrated by the spreading of resistant bacteria from livestock, landfills and sewage treatment facilities to wild animals [19,63,64]. Although food-producing domestic animals cause most human infections, under certain circumstances humans may contract *Salmonella* directly or indirectly from wildlife, especially if they come in contact with wildlife, wildlife habitats or consume game meat or plant-derived foods contaminated with wildlife excrement.

In rare cases, the occurrence of wildlife isolates in epidemiological outbreak investigation can indicate a wildlife origin. This is especially true for wildlife-adapted sequence types, such as the hedgehog-adapted *S.* Enteritidis ST183, which has been found to cause Salmonellosis in children, who may come in contact with hedgehog and hedgehog faeces during outdoor play [6,65]. Another example is the monophasic *S.* Typhimurium var. Copenhagen 4, 12:–:1, 2 of phage type DT40 [5], associated with songbirds or *S.* Choleraesuis ST145, which in Europe is linked to a reservoir in wild boar.

In most cases however, wildlife isolates in outbreak clusters reflect the proximity of human and wildlife coexistence. They are indicative of the adaptive behavior of many wildlife animals and birds, which have learned to utilize food sources provided by humans (household waste, composting sites, landfills, sewage/wastewater) and thus acquire *Salmonella* in the process. Wildlife isolates linked to outbreak clusters may also signal that outbreak-related clones already circulate widely in the environment. Overall, we conclude that occurrence of those isolates might or might not be considered for tracing analyses, depending on other epidemiological information available and on the extent of further livestock-associated isolates available for the analysis with higher likelihood contributing to human cases.

#### Limitations of This Study

Further sampling and sequencing is needed to confirm some of the results found in this study. Due to the sample selection strategy employed in this study (a combination of convenience sample and specifically selected isolates), there is a potential for spatial or temporal biases. Furthermore, data may be biased towards certain serovars, as all isolates are part of a large collection of isolates obtained for the purpose of *Salmonella* surveillance, which specifically targets serovars relevant for human and farm-animal health.

## Figures and Tables

**Table 1:** Overview of AMR genes detected from the sequence data of the *Salmonella enterica* wildlife isolates investigated in this study.

Serovar	ST	Isolates	wildlife Species	*n* AMR Genes	*n* AMR Class	AMR Class							
						Aminoglycoside	Beta-Lactam	Fluoroquinolone	Folate Pathway Antagonist	Fosfomycin	Macrolide	Phenicol	Tetracycline
*S.* Agona	13	13-SA01988-0,	common pigeon, common buzzard	1	1					*fosA7.2*			
		18-SA01710-S2											
*S.* Choleraesuis	145	20-SA00097-0,	wild boar	1	1			*gyrA_S83F*					
		20-SA00507-0,											
		20-SA01576-0											
*S.* Choleraesuis	145	20-SA00100-0	wild boar	2	1	*aph(3’’)-Ib; aph(6)-Id*							
*S.* Choleraesuis	145	16-SA01752-0, 16-SA02866-0, 18-SA00001-0, 20-SA00092-0, 20-SA00387-0, 20-SA00474-0, 20-SA00531-0, 20-SA00630-0, 20-SA00989-0, 20-SA01308-0, 20-SA02093-0	wild boar, red fox	3	2	*aph(3’’)-Ib; aph(6)-Id*			*sul2*				
*S.* Derby	40	18-SA01773-0	rook	4	3				*sul2*	*fosA7.3*	*mef(C); mph(G)*		
*S.* Derby	40	19-SA00751-0	mute swan	5	4	*aph(3’’)-Ib;aph(6)-Id*			*sul2*	*fosA7.3*		*floR*	
*S.* Enteritidis	11	17-SA00957-0	red fox	1	1			*gyrA_D87Y*					
*S.* Enteritidis	11	18-SA02674-0,	red fox	1	1			*gyrA_S83Y*					
		20-SA02382-0											
I 4,[5],12:i:-	34	19-SA00203-0	wild boar	1	1								*tet(B)*
I 4,[5],12:i:-	34	15-SA01939-0, 16-SA01483-0	roe deer, mallard	4	3	*aph(3’’)-Ib;aph(6)-Id*	*bla*_TEM-1_		*sul2*				
I 4,[5],12:i:-	34	12-03646-0,	Eurasian wolf, European starling, owl, red fox	5	4	*aph(3’’)-Ib; aph(6)-Id*	*bla*_TEM-1_		*sul2*				*tet(B)*
		15-SA02810-0,											
		19-SA01712-0,											
		19-SA01791-0											
*S.* Mbandaka	413	11-01873	European quail	6	4	*aph(3’’)-Ib; aph(6)-Id*	*bla*_TEM-1_		*sul2; dfrA14*				*tet(A)*
*S.* Typhimurium	19	08-00085-0,	roe deer, red fox	2	2	*aadA2*			*sul1*				
		08-03273-0											
*S.* Typhimurium	19	15-SA01093-0	common raven	3	3	*aadA2*		*gyrA_D87N*	*sul1*				
*S.* Typhimurium	19	16-SA00820-0	white stork	3	3		*bla*_CARB-2_	*gyrA_D87N*	*sul1*				
*S.* Typhimurium	19	09-01709-0,	red fox	5	5	*aadA2*	*bla*_CARB-2_		*sul1*			*floR*	*tet(G)*
		17-SA00138-0											
*S.* Typhimurium	128	20-SA01154-0	common pigeon	3	2		*bla*_TEM-1_		*sul2; dfrA1*				

blue = predicted located on the chromosome, yellow = predicted located on a plasmid.

**Table 2:** Isolates for joint cgMLST.

Dataset	Description	Collected By	Matrix	Isolation Years	*n*	SEN	SCS	STM
wildlife samples	isolates from wildlife animals (this study)	NRL *Salmonella* (BfR)	non-human	2002–2020	233	73	98	62
NRL database	isolates from food and farm animals collected for routine surveillance by the NRL *Salmonella*	NRL *Salmonella* (BfR)	non-human	2000–2021	1864	741	121	1002
GSS database	human clinical isolates collected for an ongoing real-time surveillance project [4]	NRC *Salmonella* (RKI)	human	2020–2021	1046	518	33	495
outbreak samples	human clinical samples collected for routine outbreak analysis by the NRC *Salmonella*	NRC *Salmonella* (RKI)	human	2017–2021	71	60	-	11
total					3214	1392	252	1570
human/non-human ratio					35/65%	42/58%	13/87%	32/68%

SEN = *S.* Enteritidis; SCS = *S.* Choleraesuis; STM = *S.* Typhimurium.

## Data Availability

Sequencing data for all isolates analysed in this study has been deposited in the NCBI Sequence Read Archive (SRA) under the BioProject accession numbers PRJEB31846 and PRJNA742494.

## Supplementary Materials

The following are available online at https://www.mdpi.com/article/10.3390/microorganisms9091911/s1. File S1 Metadata of the analysed *Salmonella enterica* wildlife isolates. File S2 Metadata, sequence quality assessment and characterisation results of the included *Salmonella enterica* wildlife isolates from other international studies. File S3 Results of the sequence quality assessment obtained with the AQUAMIS pipeline for the *Salmonella enterica* wildlife isolates analysed in this study. File S4 Characterisation results obtained with the Bakcharak pipeline for *Salmonella enterica* wildlife isolates analysed in this study. File S5 SNP distance matrices for selected serovars calculated with the snippySnake pipeline. File S6 SNP and cgMLST allele distance matrices of the mixed-matrices clusters.

## Abbreviations

AMR	antimicrobial resistance
AD	allele difference
cgMLST	core genome multilocus sequence typing
BfR	German Federal Institute for Risk Assessment
MLST	multilocus sequence typing
PCR	polymerase chain reaction
PT	phage type
RaxML	Randomized Axelerated Maximum Likelihood
RDNC	reacts with phages but does not conform with definite or provisorial types
RKI	Robert Koch Institute
MIC	Minimum inhibitory concentration
S	Salmonella enterica subsp. enterica
ST	sequence type
SNP	single nucleotide polymorphism
WGS	whole genome sequencing
NRL	*Salmonella* German National Reference Laboratory (NRL) for *Salmonella*
NRC	*Salmonella* German National Reference Center (NRC) for *Salmonella*
